# The Cunningham Panel: concerns remain

**DOI:** 10.1038/s41398-019-0562-y

**Published:** 2019-09-10

**Authors:** Susanne Bejerot, Albin Klang, Eva Hesselmark

**Affiliations:** 1 School of Medical Sciences, Örebro University, Örebro, Sweden; 2 University Health Care Research Center, Faculty of Medicine and Health, Örebro University, Örebro, Sweden; 3 Center for Psychiatry Research, Department of clinical neuroscience, Karolinska Institutet, Solna, Sweden; 4 Stockholm Health Care Services, Stockholm County Council, Stockholm, Sweden

**Keywords:** Autism spectrum disorders, Predictive markers

Dear Editor,

We thank the authors of the Connery paper^1^ for their response^2^ on the reliability of the Cunningham Panel^3^. The panel is developed and marketed by Moleculera Labs as a diagnostic test for pediatric acute-onset neuropsychiatric syndrome (PANS) and pediatric autoimmune neuropsychiatric disorder associated with streptococcus (PANDAS). Here we address some misconceptions raised by the authors and present new data.

First, the 21 healthy controls (median age 15 years) tested with the Cunningham Panel in our study were indeed healthy^4^. None had ever been diagnosed with any psychiatric, motor, or autoimmune disorder^4^. It is correct that we did not investigate previous infections or a “family history of psychiatric, autoimmune, or movement disorder”. Notably, Moleculera does not warn clinicians that these factors may affect the results of the Cunningham Panel.

Second, we are criticized for using invalid serum collection tubes in the healthy controls and the retest part of our study. Moleculera recommends glass tubes with no additives for serum collection. At the time of our study, the instructions from the company that marketed the panel in Europe (Wieslab) stated that blood should be drawn in serum tubes, with or without a separator gel (i.e., Gold Top or Red Top tubes), contrary to Moleculera’s instructions. Tubes with a serum separator gel are regarded as interchangeable with the tubes with no additive when measuring many antibodies, according to the tube manufacturer^5^. Consequently, we have questioned whether the tubes used in our study affect the Cunningham Panel results^4,5^. The reader should note that the main analysis of diagnostic accuracy was made using Cunningham Panel tests that were ordered and paid for by the patient’s treating physicians, who presumably followed Wieslab’s instructions, which included plastic tubes and gold top tubes^4^.

Although the Cunningham Panel may predict response to intravenous immunoglobulin (IVIG), this was not the case among our participants^4,6,7^. We have made a post hoc analysis including 12 patients from our dataset who had been tested with the panel prior to treatment with IVIG (2 adults, 10 children)^6^. Five had confirmed PANS and 7 suspected but not confirmed PANS. All had elevated Ca^2+^/calmodulin-dependent protein kinase II (CaMKII) values. Dopamine receptor D2 antibody results were available for 9 patients. In total, two patients had negative Cunningham Panel results when using the definition that both CaMKII and at least one antibody titer must be positive. One patient rated “no change” as response to IVIG treatment and one rated to be “much improved.” None of the Cunningham Panel analytes or the ratio between D2 and D1 antibodies predicted treatment outcome in our dataset.

Furthermore, we have compared CaMKII values between four different groups, which have been previously described: patients with confirmed PANS (*n* = 23, missing = 5)^4,7^, suspected but not confirmed PANS (*n* = 27, missing = 2)^4,7^, psychiatric controls (n = 24, missing = 8)^4,7^, and healthy controls (*n* = 21, missing = 0)^4^. All samples in this analysis were drawn at the time of our study using Gold Top tubes, not recommended by Moleculera. CaMKII values did not differ between groups. Healthy controls had higher values of anti-Lysogangioside and anti-β-tubulin antibodies than participants with confirmed PANS (Fig. 1). However, these tests were not taken on clinical indication, thus some participants may have been in remission at the time of this second testing^4^.

**Fig. 1:**
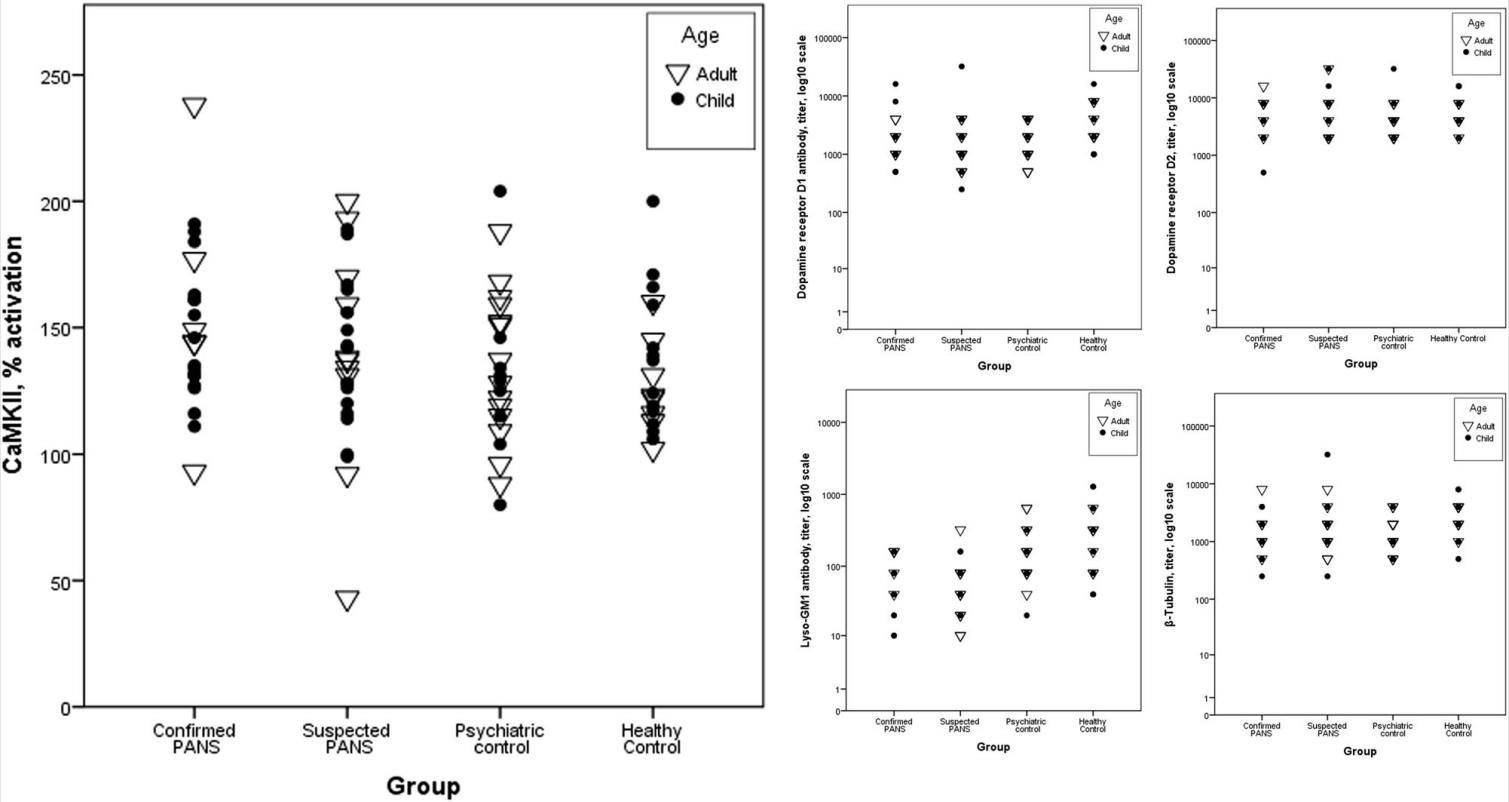
Cunningham Panel values of all patients included in our data collection. The healthy control group has been previously published^4^. These samples were all taken at the time of our study in plastic tubes with a serum separator gel (i.e., Gold Top tubes). Group distributions were compared using Kruskal–Wallis test. Post hoc analysis of medians between groups was made using Mann–Whitney test. There was no difference in CaMKII activation or dopamine receptor D2 antibody between any of the groups. Healthy controls had higher dopamine receptor D2 antibody (*p* = 0.23), Lyso GM1 antibody (*p* < 0.01), and β-tubulin antibody (*p* < 0.01) than the confirmed PANS group. Removing participants who had been treated with IVIG did not change results. Adults had higher Lyso-GM1 antibodies than children, but all other analytes were independent of age

Lastly, we have published a case report of a young woman with PANDAS^8^. She was tested with the Cunningham Panel three times resulting in both positive (132%) and negative values (99% and 109%) of CaMKII^8^.

We are concerned that Moleculera base their threshold level of positivity for CaMKII on a small sample comprising 31 non-PANDAS children of which 5 were normal human sera and 17 PANDAS cases^9^. Intriguingly, the CaMKII values of the normal children fully overlap with those of the PANDAS cases^9^.

In conclusion, concerns remain regarding the reliability of the Cunningham Panel. We advise Moleculera to publish a larger sample of healthy controls, to investigate the diagnostic and predictive value of the Panel, and to make a comparison study of different serum sampling tubes. Desperate parents pay to get the Cunningham Panel test in order to confirm that their child has a treatable disease. Most of them are satisfied with the test results as CaMKII is frequently elevated. The Cunningham Panel should only be recommended for research purposes, until further evaluations of the clinical utility are published.

## References

[1] Connery K (2018). Intravenous immunoglobulin for the treatment of autoimmune encephalopathy in children with autism. Transl. Psychiatry.

[2] Frye RE, Shimasaki C (2019). Reliability of the Cunningham Panel. Transl. Psychiatry.

[3] Bejerot S, Hesselmark E (2019). The Cunningham Panel is an unreliable biological measure. Transl. Psychiatry.

[4] Hesselmark E, Bejerot S (2017). Biomarkers for diagnosis of pediatric acute neuropsychiatric syndrome (PANS)—sensitivity and specificity of the Cunningham Panel. J. Neuroimmunol..

[5] Hesselmark E, Bejerot S (2017). Corrigendum to Biomarkers for diagnosis of pediatric acute neuropsychiatric syndrome (PANS)—sensitivity and specificity of the Cunningham Panel [*J. Neuroimmunol*. **312**, 31–37 (2017)]. J. Neuroimmunol..

[6] Hesselmark, E. & Bejerot, S. Patient satisfaction and treatments offered to Swedish patients with suspected pediatric acute-onset neuropsychiatric syndrome and pediatric autoimmune neuropsychiatric disorders associated with streptococcal infections. *J. Child Adolesc. Psychopharmacol*. 10.1089/cap.2018.0141 (2019). 10.1089/cap.2018.0141 PMC6786336 31009235

[7] Hesselmark E, Bejerot S (2019). Clinical features of paediatric acute-onset neuropsychiatric syndrome: findings from a case–control study. BJPsych Open.

[8] Bejerot S (2018). Neuromyelitis optica spectrum disorder with increased aquaporin-4 microparticles prior to autoantibodies in cerebrospinal fluid: a case report of a PANDAS patient. J. Med. Case Rep..

[9] Kirvan CA, Swedo SE, Snider LA, Cunningham MW (2006). Antibody-mediated neuronal cell signaling in behavior and movement disorders. J. Neuroimmunol..

